# Evaluation of oxalic acid extraction and quantification methods in the different purslane (*Portulaca oleracea* L.) matrices and spinach (*Spinacea oleracea*)

**DOI:** 10.1016/j.mex.2024.102863

**Published:** 2024-07-14

**Authors:** Pâmela Gomes de Souza, Juliana Azevedo Lima Pallone, Eduardo Adilson Orlando, Augusto César Costa-Santos, Ellen Mayra Menezes Ayres, Amauri Rosenthal, Anderson Junger Teodoro

**Affiliations:** a Laboratory of Food Analysis, Graduate Program in Food Nutrition, Fluminense Federal University, Rua Mário Santana Braga, 30 - Niteroi, RJ, Brazil; b Laboratory of Food Analysis by Spectroscopy, Faculty of Food Engineering, State University of Campinas, Rua Monteiro Lobato, 80 Barão Geraldo, Campinas, SP, Brasil; c Sensory and Consumer Science Laboratory, Postgraduate Program in Food and Nutrition, Federal University of the State of Rio de Janeiro Laboratorio, Av. Pasteur -Urca, 29, Rio de Janeiro, Brazil; d Embrapa Food Technology, Av. das América, 29501, Guaratiba, Rio de Janeiro, RJ, Brazil

**Keywords:** Oxalic acid, Antinutrients, Extraction, FAAS, UV-vis spectrophotometry, Oxalic acid quantitative analysis

## Abstract

Purslane (*Portulaca oleracea*) and spinach (*Spinacea oleracea*) are species with elevated levels of oxalic acid, an antinutrient that interferes in the bioaccessibility of minerals such as calcium and iron. Evaluating methods to determine oxalic acid content with reduced matrix interference, such as employing Flame Atomic Absorption Spectrometry (FAAS), can enhance the specificity of determinations. The different matrices of purslane (whole plant, leaves, and juice) and spinach (whole plant) were tested using three extraction methods (M1, M2, and M3). The oxalic acid content was evaluated by UV-vis spectrophotometry and FAAS (Flame Atomic Absorption Spectrometry). The absence of the precipitation step in M1 resulted in high levels of oxalic acid in the investigated matrices. The quantification of oxalic acid by FAAS for M2 (6M HCl for 1 hour at 100°C) and M3 (0.25N HCl for 15 minutes at 100°C) in the samples of purslane leaves and spinach whole plants yielded statistically similar results. However, the analysis by UV-vis spectrophotometry for M2 and M3 showed significant discrepancies in all evaluated samples, suggesting interference from colored compounds in the food matrix.

• Comparison of methods of extraction
• Comparison of UV-vis spectrophotometer and FAAS in the quantification of oxalic acid
• Analysis of antinutrients in plant matrices

## Graphical Abstract

**Figure:**
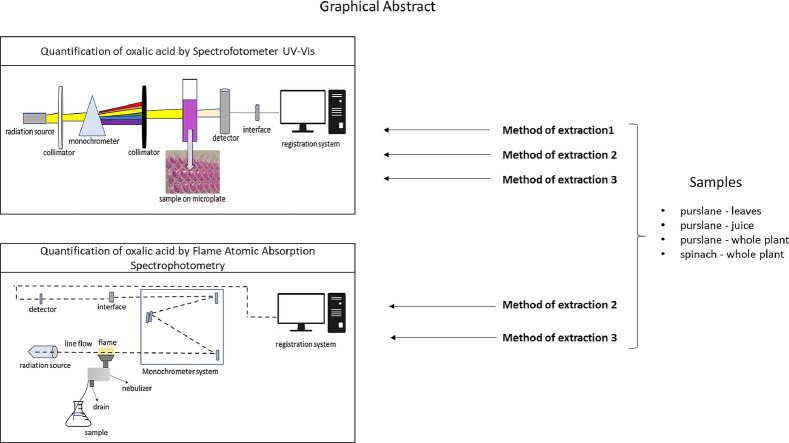

Specifications table

**Table:** 

Subject area:	Chemistry
More specific subject area:	Quantitative analyses
Method name:	Oxalic acid quantitative analysis
Name and reference of original method:	Naik et al., (2014) [12]; AOAC (1990) [8]; Adeniyi et al., (2009) [13].
Resource availability:	NA

## Background

Oxalic acid is found in large quantities in vegetables such as spinach and purslane. This antinutrient can form soluble oxalates (complexes with sodium, potassium, and ammonium) and insoluble oxalates (complexes with calcium, magnesium, and iron) [1]. When complexed with calcium, oxalic acid can form calcium oxalate crystals in the kidneys, reducing the bioavailability of this mineral [2]. The recommended daily intake of oxalic acid is 50-200 mg/day, with the minimum lethal dose for adults being 5 grams. [[3], [4], [5]]. Some techniques for reducing oxalic acid in food are already known and employed, such as cooking and bleaching [5]. However, juices are commonly produced without prior heat treatment of the vegetables, resulting in potentially high oxalic acid content in the final product [6]. The most commonly methods of analysis to quantify oxalic acid in food matrices are titration, High Performance Liquid Chromatography (HPLC) and UV-vis spectrophotometry [2,[7], [8], [9], [10]]. UV-vis spectrophotometry has numerous advantages, such as being an inexpensive method for quantifying a variety of organic compounds. This method is also widely used coupled with HPLC [11]. For UV-vis spectrophotometry, chromophore compounds can absorb within the same range as the analyte of interest when the method is used without coupling with complementary techniques for characterization. The indirect determination of oxalic acid in canned vegetables through calcium quantification using the Atomic Absorption Spectrometry (AAS) is described in AOAC (1990). FAAS is an Atomic Absorption Spectrometry that utilizes a flame as an atomizer. This technique can be an alternative to other established methods for determining oxalic acid amounts, with fewer interferences from chromophore compounds. This study aimed to compare three extraction methods, two quantification techniques (FAAS and UV-Vis spectrophotometric) and also investigate the inclusion of the precipitation step to form and separate calcium oxalate.

## Method details

### Sample preparation

#### Method of extraction 1 (M1)

This method was performed according to the reference [12]. Triplicate samples of 0.5g of sample (freeze-dried) or 2g (liquid) were weighed and digested with 30mL of 0.25N HCl for 15 min at 100°C. After cooling to room temperature, the volume was adjusted to 50 mL with 0.25 N HCl. The precipitation step was not performed in M1.

#### Method of Extraction 2 (M2)

The M2 was performed according to reference [13], and with adaptations. For extraction, triplicate samples of 0.5 g (freeze-dried) or 2 g (liquid) were weighed and digested with 10mL of 6M HCl for 1 hour at 100 °C. The digested solution was completed to 100 ml with ultrapure water, filtered and cooled. For precipitate calcium oxalate, concentrated NH_4_OH was dripped before the color changed from salmon pink to faint yellow, and the pH was adjusted to 4-4.5, monitored using methyl red indicator for visualization of the color change. The precipitation step consisted of adding 15 mL of 5 % CaCl_2_, followed by centrifugation of the suspension (2500 rpm/5 min) and decantation of the supernatant. The precipitate was dissolved in 10 ml of 20 % H_2_SO_4_ and completed to 50 ml with ultrapure H_2_O.

#### Method of Extraction 3 (M3)

For executing M3, the extraction followed the methodology described in reference [12], with adaptations. Triplicate samples of 0.5 g (freeze-dried) or 2 g (liquid) were weighed and digested with 30 mL of 0.25N HCl for 15 min at 100 °C. The precipitation step was executed as described by Adeniyi et al., (2009), detailed in M2. The precipitate was dissolved in 10 ml of 20 % H_2_SO_4_. The resulting precipitate (calcium oxalate) was adjusted to 50 ml with ultrapure 0.25N HCl.

#### Quantification of oxalic acid by UV-vis spectrophotometry

The UV-vis spectrophotometer (Fluo Star Omega, BMG Labtech, Ortenberg, Germany) was used to measure light absorption at a wavelength of 528 nm. The method was based on the oxidation reaction of oxalic acid by KMnO_4_, adapted of Naik et al. (2014). For each sample (M1 and M2) or the reagent blank (containing only water), 25 µL of the extracts were mixed with 125 µl of 2N H_2_SO4 and 50 µl of 0.003M KMnO_4_. The mixtures were incubated at 27 °C, for 10 min.

**Figure:**
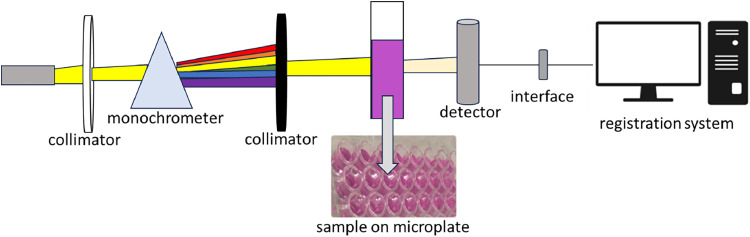

#### Standard oxalic acid solution for analysis in UV-vis spectrophotometry

The potassium permanganate solution was prepared, and as the concentration of oxalic acid increased, the absorbance decreased. Different concentrations of the oxalic acid standard curve were prepared from the 1mg/mL stock solution, ranging from 0.1 to 0.9 mg/mL.

#### Quantification of oxalic acid by Flame Atomic Absorption Spectrophotometry (FAAS)

The indirect measurement of oxalic acid was evaluated by quantifying the total calcium content of the samples using a flame atomic absorption spectrometer (Analyst 200, Perkin-Elmer, Norwalk, USA). The determination of Ca was conducted through the specific wavelength of a hollow cathode lamp set at 423 nm, in a flow rate of 2.7/0.6 L/min of air/acetylene gas input. A 0.5 % lanthanum oxide solution was added to the final sample solution to prevent interferences of phosphate ions. The same procedure was executed for the analytical blanks, used for quantifying extracts M2 and M3. A standard solution of 1000 mg/L Ca was used to prepare the analytical curve, distributed in five equidistant concentrations ranging from 0.5 to 5.0 mg/L.

**Figure:**
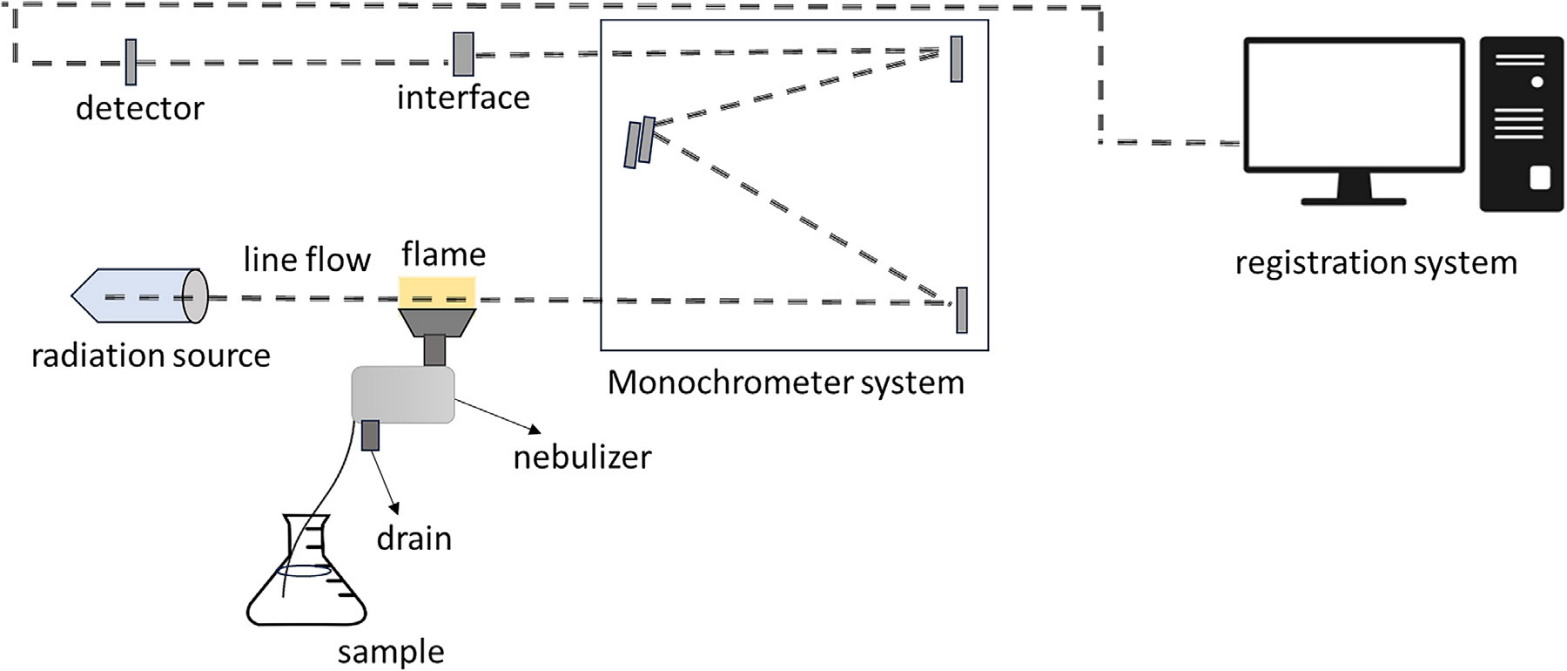

#### Standard calcium solution for analysis in FAAS

Calcium standards were prepared with lanthanum oxide 10 %, HNO_3_, and ultrapure water.

## Results and discussion

### Formula for determination of oxalic acid in the UV-vis spectrophotometry

**Fig. 1:**
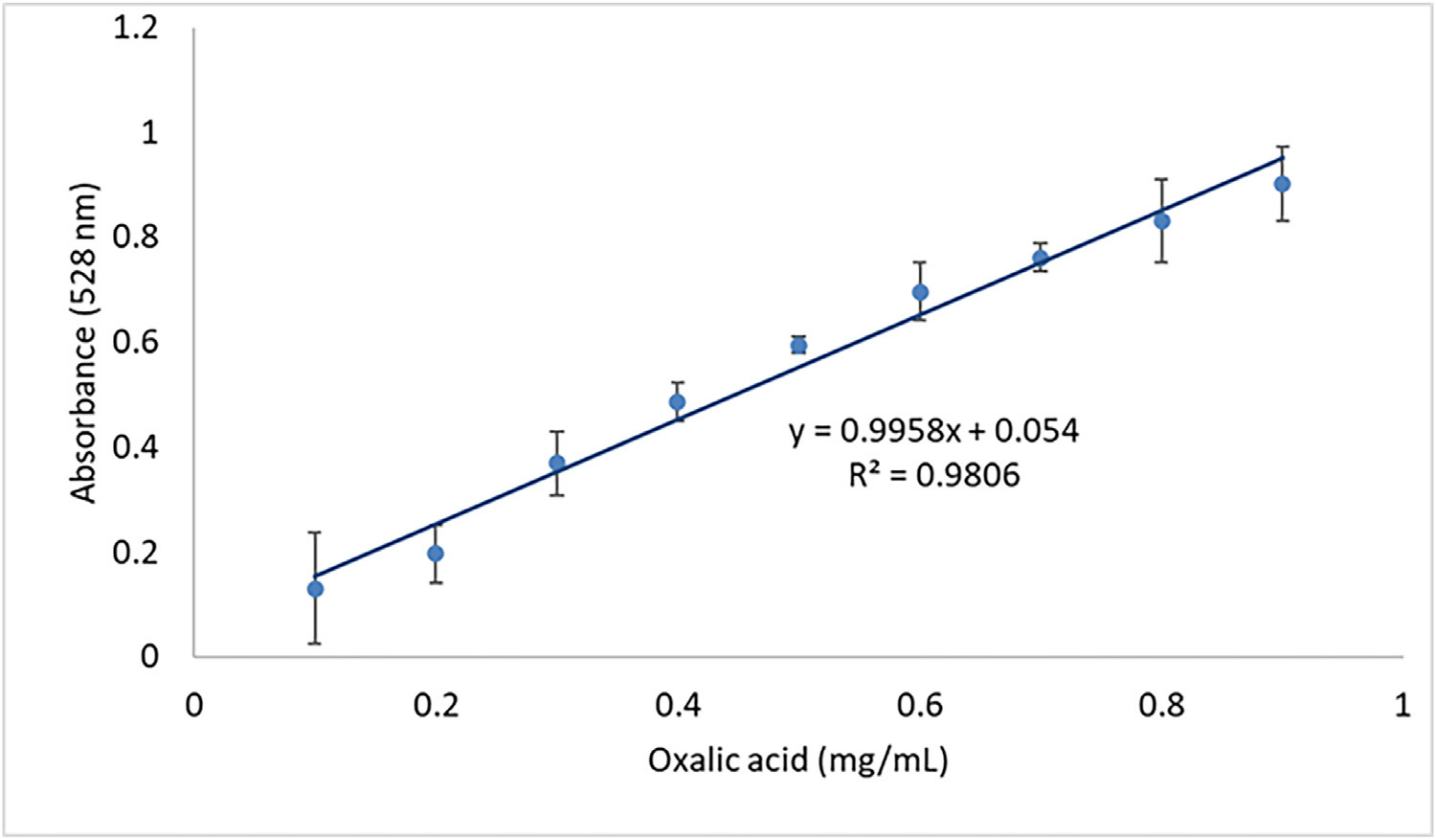
Regression line for quantification of oxalic acid in triplicate.

The final absorbance values for the standard curve were obtained using the formula (B-A) as presented in Table 1. The linear regression was obtained through the equation (y = 0.9958x + 0.054) and the determination coefficient (R2 = 0.9806) is shown in Fig. 1 (Table 2,Table 3).

**Table 1:** Calculation of the final absorbance for the development of the standard.

Concentration of oxalic acid (mg/mL)	Standard curve	
	Absorbance (A)	Final absorbance = (B-A)
0.1	1.6335	-0.1311
0.2	1.5675	-0.1971
0.3	1.3955	-0.3691
0.4	1.2785	-0.4861
0.5	1.17	-0.5946
0.6	1.0685	-0.6961
0.7	1.004	-0.7606
0.8	0.9335	-0.8311
0.9	0.8635	-0.9011

*B(reagente blank). The absorbance value obtained for B was 1.7646.

**Table 2:** M1 and M3 test in UV-vis spectrophotometry.

	Oxalic acid concentration			
	purslane - juice (g/100 g WM)	purslane - whole plant (g/100 g DM)	purslane (leaves, g/100 g dry matter)	spinach - whole plant (g/100 g DM)
M1	3.883 ± 0.121^a^	33.21 ± 0.5354^a^	32.47 ± 0.53^a^	40.11 ± 1.16^a^
M3	0.9176 ± 0.03769^b^	12.26 ± 0,091^b^	13.56 ± 0.7448^b^	13.75 ± 0.92^b^

*Different letters indicate statistically different values (p<0.05) for Tukey Test.

**Table 3:** Comparison between UV-vis spectrophotometry and FAAS.

	Oxalic acid concentration			
	purslane - juice (g/100g wet matter)	purslane - whole plant (g/100g dry matter)	purslane - leaves (g/100g dry matter)	spinach - whole plant (g/100g dry matter)
M2 (UV-vis spectrophotometry spectrophotometer)	0,67 ± 0,011^a^	9,51 ± 0,75^a^	28,73 ± 2,30^a^	22,01 ± 1,71^a^
M3 (UV-vis spectrophotometry)	0,91 ± 0,037^b^	11.96 ± 0,09^b^	13.01 ± 0,74^b^	11.87 ± 0,82^b^
M2 (FAAS)	0,24 ± 0,01^c^	1,62 ± 0,11^c^	7,613 ± 0,73^c^	10,40 ± 0,12^c^
M3 (FAAS)	0,37 ± 0,072^d^	5,55 ± 0,49^d^	6,56 ± 0,28^c^	10,52 ± 0,40^c^

*Different letters indicate statistically different values (*p* < 0.05) for Tukey Test.

To calculate the final absorbance of the sample (FA), the absorbance of the sample (S) was subtracted from the absorbance of the reagent blank (B). Thus, FA = B-S. For determining the linear regression equation, the analyte concentration was obtained by the following formula:

y=ax+b

FA = 0.9958x + 0.0054, where x represents the final concentration of the analyte in mg/mL. To express the result in grams (g) of weighed sample, the concentration must be multiplied by the final volume of the extract (50 mL). This calculation yields the amount of analyte present in the sample. Afterward, simply express the result as a percentage for 100g of the sample ( %).

#### Formula for indirect calculation of oxalic acid in the FAAS

**Fig. 2:**
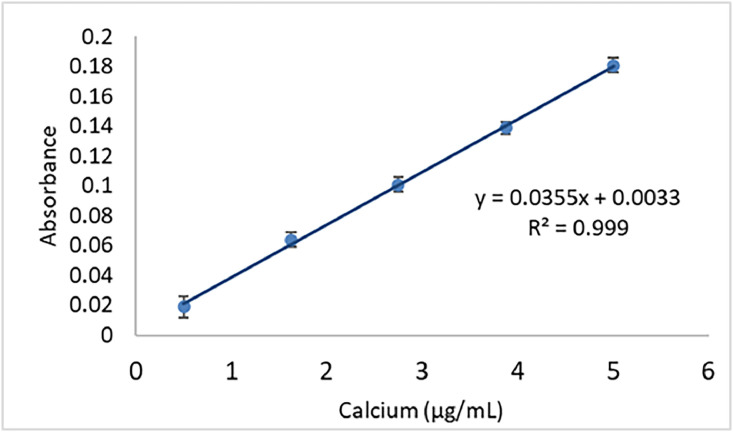
Regression line for quantification of calcium in triplicate.

The linear regression equation for calcium quantification through the standard curve is shown in Fig. 2. The final absorbance of the sample (FA) can be calculated by absorbance readings of the reagent blank (B) subtracted by the absorbance of the sample (S).

Therefore, FA = S-B.

The formula used to calculate the oxalic acid content was determined indirectly through the quantification of calcium in the sample, according to the AOAC method (1990), with adaptations:

$$\frac{mg\;oxalic\;acid/100g=\mu gCa/mlx\left[FC\;x\;\left(final\;volume\;x\;FD\right)\;X\;100\right]}{sample\;weight\;\left(g\right)}$$

Where:

FC = 2.246/1000 (conversion of micrograms calcium to mg oxalic acid)

FD = dilution factor

**Fig. 3:**
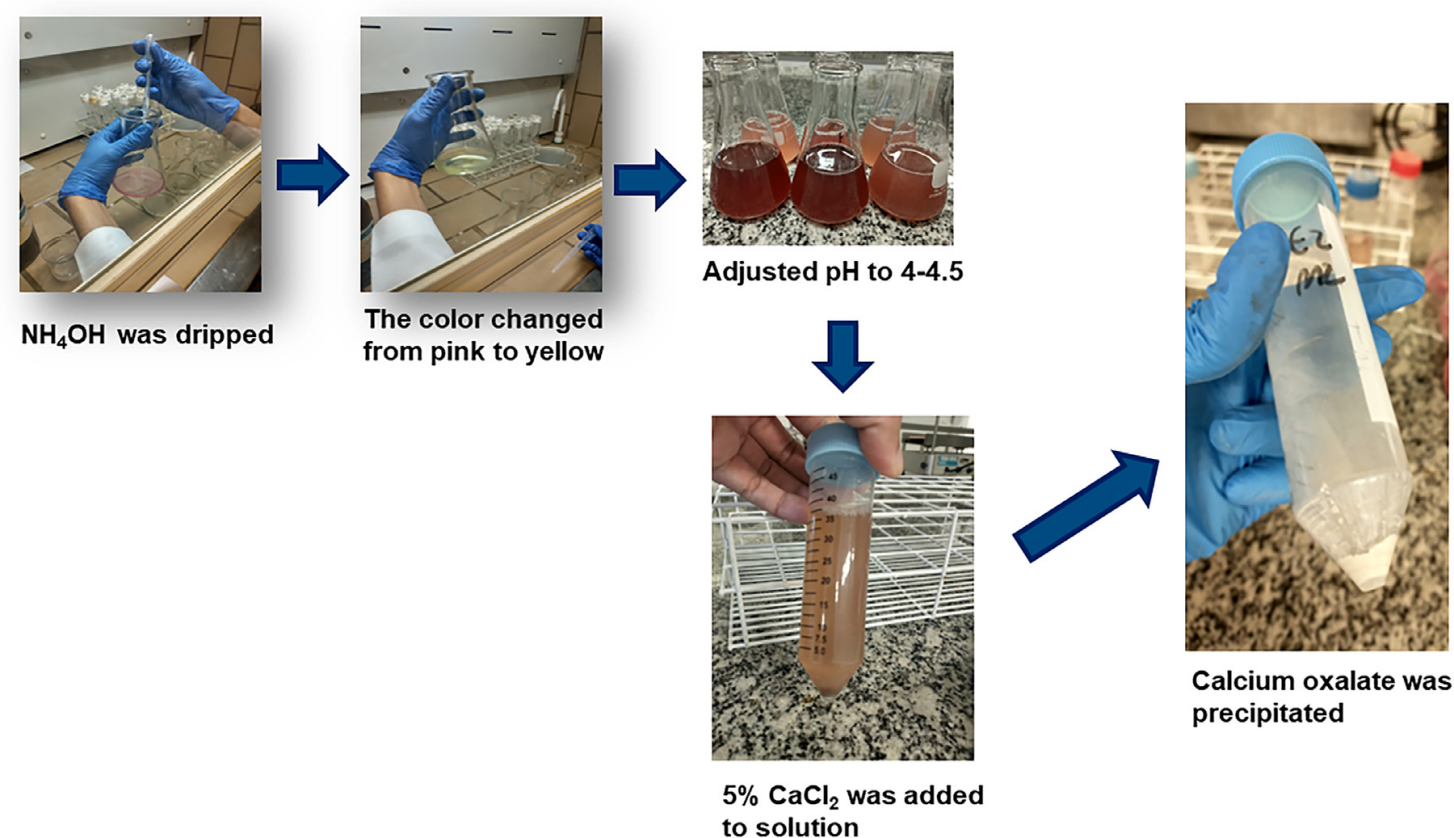
Precipitation of calcium oxalate.

The comparison between the methods using the UV-vis spectrophotometry revealed that M1 (without the precipitation step) showed higher oxalic acid content compared to M3. UV-vis spectrophotometry operates by selecting a wavelength where the substance of interest exhibits maximum absorption. However, other substances may absorb at the same wavelength, potentially causing errors in the result. Therefore, precipitation is employed to isolate the compound of interest (calcium oxalate) and reduce analytical interferents. The formation of calcium oxalate during the precipitation step is illustrated in Fig. 3.

Naik et al., (2014) evaluated the oxalic acid content in purslane leaves using UV-vis spectrophotometry (M1). The coefficient of determination (R^2^) for the oxalic acid standard curve reported by the authors was 0.983, close to the value of 0.9806 observed in the present study. Additionally, the authors found oxalic acid levels of 6.36 g/100g in dry weight for *Portulaca oleracea*. They also measured oxalic acid by titration with KMnO_4_, obtaining values of 6.34 g/100g, similar to those obtained by UV-vis spectrophotometry. The oxalic acid levels found by Naik et al. (2014) were lower than those found in M1 of this study. This difference may be attributed to variations in the location where the purslane was harvested. Factors such as climate, temperature, humidity and the stage of maturation can influence the composition of the plant.

In the UV-vis spectrophotometry analysis, M3 showed higher contents for the juice and purslane–whole plant, but this trend was not observed for purslane-leaves and spinach-whole plant, where M2 demonstrated significantly higher contents. Each matrix can present many differences in its constituents, which can result in interference during spectrophotometric analysis. One of the disadvantages of using the Uv-vis spectrophotometry is the interference of colored compounds present in the sample [14].

Regarding the method employed by Adeniyi et al. (2009), oxalate extraction from Nigerian food samples involved a digestion step (10 mL 6M HCl for 1 hour), followed by precipitation as detailed in M2 of this study. However, oxalate quantification was performed using titration with standardized KMnO_4_ solution until reaching a light pink coloration. According to the study, leaves of *Amaranthus* sp. exhibited 91.9 mg/100g of fresh weight. Instrumental techniques offer higher sensitivity for quantifying target analytes compared to titration methods, leading many studies to utilize more advanced techniques such as UV-vis spectrophotometry, Flame Atomic Absorption Spectrometry (FAAS), and High-Performance Liquid Chromatography (HPLC).

The quantification technique of oxalic acid using HPLC has been widely employed in the literature [1,6,[15], [16], [17], [18], [19], [20], [21]]. Extraction parameters (pH, temperature, and time) for determining oxalic acid in spinach via HPLC were investigated by Kusuma et al. (2016), who found levels ranging from 424.95 to 856.57 mg/100g of fresh matter sample. Savage et al. (2000) also determined total oxalate content in spinach using the HPLC method, reporting levels of 329.6 mg/100g of fresh weight sample. Elevated levels of oxalic acid in spinach samples were also found in the current study (10.52 – 22.01 mg/100g of dry matter) for M2 and M3. The quantification of oxalic acid by AAS was described in AOAC (1990), while the determination of this antinutrient content by FAAS was conducted in the present study. As for the analysis by FAAS, the M3 presented higher contents for the purslane-juice and purslane-whole plant samples, not differing significantly from the M2 when compared with purslane-leaves and spinach-whole plant. The results of the FAAS analysis indicate a minor difference between the evaluated extraction methods, suggesting less interference when using this technique compared to UV-vis spectrophotometry.

In addition, M3 can be a better option due to the use of more dilute acid and shorter extraction time compared to M2, making it safer for analysts and contributing to Green Chemistry.

## Conclusions

The comparison of oxalic acid extraction and quantification methods described in the literature and modified in this research can help to obtain more reliable results on the contents of these antinutrients in matrices of purslane (*Portulaca oleracea* L.) and spinach (*Spinacea oleracea*). The precipitation step was essential in reducing the influence of matrix interferents during oxalic acid quantification. Extraction using diluted acid (0.25N HCl) for a short time (15 min) resulted in lower result fluctuations. Additionally, the absence of interference from colored compounds in matrices is an advantage of using FAAS. Therefore, employing more dilute acid for a shorter duration and quantifying by FAAS, as demonstrated in M3, resulted in better performance for determining oxalic acid content, besides contributing to Green Chemistry.

## CRediT authorship contribution statement

**Pâmela Gomes de Souza:** Conceptualization, Methodology, Software, Data curation, Writing – original draft. **Juliana Azevedo Lima Pallone:** Supervision, Writing – review & editing. **Eduardo Adilson Orlando:** Methodology, Investigation, Data curation. **Augusto César Costa-Santos:** Writing – review & editing. **Ellen Mayra Menezes Ayres:** Writing – review & editing. **Amauri Rosenthal:** Visualization, Writing – review & editing. **Anderson Junger Teodoro:** Supervision, Writing – review & editing.

## Declaration of competing interest

The authors declare that they have no known competing financial interests or personal relationships that could have appeared to influence the work reported in this paper.

## Data Availability

No data was used for the research described in the article.
